# Stable isotopes measurements reveal dual carbon pools contributing to organic matter enrichment in marine aerosol

**DOI:** 10.1038/srep36675

**Published:** 2016-11-07

**Authors:** Darius Ceburnis, Agne Masalaite, Jurgita Ovadnevaite, Andrius Garbaras, Vidmantas Remeikis, Willy Maenhaut, Magda Claeys, Jean Sciare, Dominique Baisnée, Colin D. O’Dowd

**Affiliations:** 1 School of Physics & Ryan Institute’s Centre for Climate and Air Pollution Studies, National University of Ireland Galway, Galway, Ireland; 2 Department of Nuclear Research, Center for Physical Sciences and Technology, Vilnius, Lithuania; 3 Department of Analytical Chemistry, Ghent University, Gent, Belgium; 4 Department of Pharmaceutical Sciences, University of Antwerp (Campus Drie Eiken), Antwerp, Belgium; 5 Laboratoire des Sciences du Climat et de l’Environnement, CNRS-CEA-UVSQ, Gif-sur-Yvette, France; 6 Energy Environment Water Research Center (EEWRC), The Cyprus Institute, Nicosia, Cyprus

## Abstract

Stable carbon isotope ratios in marine aerosol collected over the Southern Indian Ocean revealed δ^13^C values ranging from −20.0‰ to −28.2‰. The isotope ratios exhibited a strong correlation with the fractional organic matter (OM) enrichment in sea spray aerosol. The base-level isotope ratio of −20.0‰ is characteristic of an aged Dissolved Organic Matter (DOM) pool contributing a relatively homogeneous background level of DOM to oceanic waters. The range of isotope ratios, extending down to −28.2‰, is characteristic of more variable, stronger, and fresher Particulate Organic Matter (POM) pool driven by trophic level interactions. We present a conceptual dual-pool POM-DOM model which comprises a ‘young’ and variable POM pool which dominates enrichment in sea-spray and an ‘aged’ but invariant DOM pool which is, ultimately, an aged end-product of processed ‘fresh’ POM. This model is harmonious with the preferential enrichment of fresh colloidal and nano-gel lipid-like particulate matter in sea spray particles and the observed depleted δ^13^C ratio resulting from isotope equilibrium fractionation coupled with enhanced plankton photosynthesis in cold water (−2 °C to +8 °C). These results re-assert the hypothesis that OM enrichment in sea-spray is directly linked to primary production and, consequently, can have implications for climate-aerosol-cloud feedback systems.

Organic matter reservoir in the Earth’s oceans is by far the largest store of OM outcompeting both terrestrial or atmospheric reservoirs and driven by biological activity and trophic level interactions^1^. It is traditionally operationally divided into two categories, dissolved (DOM) and particulate (POM) organic matter, the former constituting by far the largest contributor to total OM in sea water. The distinction between DOM and POM is simply the OM that can pass through the most commonly used 0.5 micron filter^2^. DOM, by comparison to POM, is considered to be relatively uniform in its distribution across the world oceans with an average concentration at the surface of 70–150 μM^1^. POM is comprised of a diverse pool of species ranging from lipid-like to polysaccharide-like mixtures, being a product of phytoplankton exudates or cell debris while DOM is considered to be a processed mixture of soluble carbohydrates and humic-like substances. Chlorophyll-a has been demonstrated to be a valid proxy for OM source parameterisations^3^, but there has been a significant debate whether such a proxy can reproduce seasonal variability across all the world oceans. Long-term studies of marine aerosol organic matter spanning several years of continuous measurements^4^,^5^ revealed a profound seasonality linked to an apparent biological activity^6^, while short-term ship-born campaigns were unable to reproduce the relationship between organic matter fraction in sea spray and phytoplankton abundance parameterised by chlorophyll-a proxy^7^. Recent evidence, however, pointed towards trophic level interactions modifying the proposed organic matter and chlorophyll-a relationship and introducing a lag between peak phytoplankton and the OM fraction in sea spray^8^,^9^. Ultimately, a coupled ocean-atmosphere model using a novel framework based on a competitive Langmuir adsorption equilibrium at bubble surfaces^10^ can bridge the gap between trophic level processes and observations of organic matter in sea spray particles.

Carbon isotope analysis can be extremely useful in source apportionment of organic matter due to the unique isotopic signatures associated with anthropogenic (fossil fuel), continental (terrestrial plants) and marine sources, and is particularly effective when these sources are mixed^11^. Pioneering stable carbon measurements^12^,^13^ undertaken on a global scale revealed the presence of terrestrial and anthropogenic carbon even at remote locations, but also discovered the unique signature values of δ^13^C in unperturbed marine environment^14^,^15^,^16^. Here, we present the first systematic measurements of stable carbon isotopes along with fully resolved aerosol chemical composition of atmospheric aerosols at Amsterdam Island in the Southern Indian Ocean during an entire austral summer of 2007. Amsterdam Island is located in the middle of a vast ocean equidistant from Africa, Australia and Antarctica and slightly above the Circumpolar Antarctic Circulation boundary, making it an ideal location for studying unperturbed marine aerosol^5^,^17^. Stable carbon isotopes were measured in fine (<2.5 μm) and coarse (>2.5 μm) particulate matter samples of 5-day sampling duration from December 2006 to March 2007. The range of stable carbon isotope ratios spanned between −20.35‰ and −28.23‰, far lower than in the previous studies from the Northern Hemisphere (−20 to −24‰)^11^,^14^,^16^.

A wide range of observed stable carbon isotope ratios, specifically, the low-end values may suggest either anthropogenic OM (excluded due to remoteness of the site and extremely low BC values well documented at Amsterdam Island^5^) or fresh secondary organic aerosol (SOA) composed from unprocessed isotope-light precursors, e.g. isoprene^18^. Photochemically processed SOA isotope values would typically be isotopically enriched due to atmospheric oxidation induced isotope fractionation, however, all isotopically enriched samples contained the lowest amount of OC mass suggesting minor contribution of SOA to OM content. Coarse fraction isotope values were significantly lower than those of the fine fraction values consistent with less atmospheric processing of coarse particles due to their shorter residence time. Moreover, the organic carbon (OC) fraction in sea spray (OC_f_ = OC/(OC + SeaSalt)) plotted against stable carbon isotope ratio presented in Fig. 1, reveals, for the first time, an inverse linear relationship, both in coarse and fine fractions (r = −0.89 and r = −0.66, respectively, at P < 0.01). It is worth noting that the OC fraction at Amsterdam Island during austral summer (up to 15% in PM2.5 size fraction) was very similar to the fractional OC values over the eastern North Atlantic at Mace Head during peak biological activity period (up to 70% in PM1 size fraction)^8^,^19^ when the difference in sea salt content in PM2.5 and PM1 size fractions was taken into account^20^. Air mass back trajectory analysis presented in Suppl. Figure 1, indicated that the trajectories originating at higher latitudes with higher chlorophyll-a concentrations and lower sea surface temperatures were associated with the more negative carbon isotope ratios and, combined with the relationship in Fig. 1, necessitated the mixing of at least two isotope sources to explain the observed pattern.

A model comprising two organic matter pools is proposed to explain the observations in a manner consistent with the main biogeochemical processes. The proposed model also encapsulated the relationship between phytoplankton δ^13^C and water temperature^21^ over the sea water temperature gradient of 30 °C. A change in phytoplankton δ^13^C of about 0.5‰ per degree Centigrade (δ^13^C = 0.5T_w_ − 29) was found^21^.

Of the two organic matter pools proposed, one corresponds to a “fresh” and highly variable OM pool directly linked to phytoplankton primary exudates, which is temperature dependant, while the other corresponds to an “aged” OM pool represented by processed organic carbon in sea water with the stable δ^13^C ratio of about −19‰ as the upper limit from Fig. 1 22. This “aged” OM pool was considered to have contributed a constant amount of OM taken as the minimum OC concentration observed (40 ng m^−3^) and was in the order of 10% of the maximum OC concentration in fine particles (360 ng m^−3^). These two OM pools were combined in an isotope mixing equation:


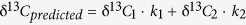


where *δ*^*13*^*C*_1_ is the isotope ratio of “fresh” OM source with *δ*^*13*^*C* ranging from −30‰ to −19‰^21^ in accordance to δ^13^C = 0.5T_w_ – 29; *δ*^*13*^*C*_*2*_ is the isotope ratio of the “aged” or processed OM source with the corresponding value of δ^13^C_2_ = −19‰^22^; *k*_*1*_ and *k*_*2*_ are the relative contributions of the two sources (*k*_*1*_ + *k*_*2*_ = *1*), depending on biological activity level, ranging from 0.1 to 0.9, respectively.

Selected sea salt concentrations (1.2 μg m^−3^, 1.7 μg m^−3^, 2.7 μg m^−3^ and 4.0 μg m^−3^) spanning the observed range of concentrations were used in the model to plot the predicted δ^13^C ratio against the fractional OC in fine particles. The results of the proposed model are summarised in Fig. 2 where the webs of coloured isolines represent the mixing of the two sources over the entire sea salt concentration range and the OC concentration range. One set of isolines was entirely outside the observed relationship range and corresponded to a low sea salt concentration resulting from calm seas. The remaining three sets of isolines overlap with the key areas of the observations, but in all cases, only the isolines corresponding to low sea surface temperatures characteristic of Circumpolar Antarctic Current (−2 °C–+8 °C) match the observed measurement points. The majority of the observed isotope values overlapped with the set of isolines corresponding to medium-to-high sea salt content related to rough sea conditions typical of high latitudes around Antarctica (40–60 degrees South) and, therefore, resulting in a slightly lower fractional OC in agreement with the proposed parameterisation of Gantt *et al*.^23^.

The results of the proposed model not only overlap with the observations, but are consistent with key biogeochemical processes in the ocean surface which are tightly linked to plankton photosynthesis and concurrent isotope fractionation processes. The more negative δ^13^C values of phytoplankton biomass at lower sea water temperature was a combined effect of equilibrium isotope fractionation (0.35‰/^o^C)^24^ and increased RUBISCO activity of phytoplankton cells growing in colder water^21^. The higher fractional OC linked to more negative δ^13^C values and corresponding low temperatures was supported by a larger lipid content produced by phytoplankton cells growing in cold water^25^. The higher lipid abundance is considered to be the key in OM enrichment in sea spray due to lipid ability to form gels, micelles and colloids^9^ characteristic feature of long-chain hydrocarbons^26^,^27^. Higher fractional OC corresponding to lower sea water temperature is consistent with the general pattern of higher primary productivity as inferred from chlorophyll-a satellite images and corresponding analysis of the dominant phytoplankton species at high latitudes in both Hemispheres^28^. The more enriched δ^13^C values (suggested by equilibrium fractionation alone) typically occur due to the buffering action of bicarbonate ions when aqueous CO_2_ is depleted around cells in intense phytoplankton blooms forcing bicarbonate to dissociate and replenish CO_2_ with the heavier carbon^29^. A slightly different slope of fine fraction relationship likely indicates a transition from one hypothesised OM pool to the other as fine fraction particles were enriched by partially processed OC, while the coarse fraction OC was more directly related to phytoplankton biomass. The proposed model elucidates previous stable carbon isotope measurements in sea spray^11^ in that the limited range of observed δ^13^C values ranged from −20‰ to −24‰ can be explained be the limited temperature range existing in the North Atlantic (+10 °C to +16 °C). All other studies conducted during the Northern Hemisphere summer^15^ or in warm subtropical waters^12^,^13^,^16^ reported marine δ^13^C values close to −20‰ corresponding to the upper limit of model values. A rather uniform fractional OM in sea spray observed at the same location^5^ during an entire year and its peak during biologically active austral summer is also consistent with the proposed model.

A schematic diagram of a proposed model and its relevance to the biogeochemical cycling of marine organic matter is presented in Fig. 3 along with basic atmospheric processes modifying airborne organic matter, both primary and secondary. Photosynthesising plankton produce isotope-light organic matter which undergoes isotope fractionation during trophic level induced OM breakdown and decomposition and is gradually becoming isotope-heavier. A mixture of the two sources ends up in sea spray (primary marine aerosol - POA) and is further isotopically fractionated in the atmosphere towards isotope-heavy end-member depending on the particle residence time. Coarse particles are considered mainly consisting of POM source while fine particles consisting of the dual pool dynamic mixture. The processed and isotope-heavy OM is deposited back to the ocean completing the cycle. The two distinct pools are coexisting dynamically, but ultimately resulting in sequestered organic matter which is isotope-light (like most fossil-fuels) and continuously recycled OM which tends to be isotope-heavy. Secondary organic aerosol (SOA) was not explicitly defined in the model, but would initially comprise of isotope-light precursors^14^ and would be isotopically similar to the POM generated at low sea water temperatures. Kinetic isotope fractionation during atmospheric ageing, e.g. cloud processing, would gradually make SOA isotope-heavier just as much as the processed primary organic matter following the same scheme towards the isotope-heavy end-member.

This rather simplified dual organic matter pool framework offers critical constrains to the ongoing efforts of biogenic sea spray source function development in global ocean-atmosphere models.

## Methods

The aerosol samples were collected at Amsterdam Island (37.48°S, 77.34°E), which is located approximately 3000 km from Antarctica and Australia and 5000 km from Africa and India. A high-volume dichotomous sampler was used to collect samples in fine (<2.5 μm AD) and coarse (>2.5 μm AD) aerosol fractions at a flow rate of 250 and 27 lpm in fine and coarse channels respectively. The sampler was located at 30 m above sea level. Quartz fibre filters were pre-fired at 550 °C for 24 h and 18 samples of 5-day duration were collected between 3^rd^ of December 2006 and 4^th^ of March 2007. All samples were frozen until the analyses.

Carbon isotope ratio (δ^13^C) measurements were performed using a stable Isotope Ratio Mass Spectrometer (IRMS) calibrated by the Vienna Pee Dee Belemnite (VPDB) standard^30^. The filters were analysed with the elemental analyzer FlashEA 1112 connected to the stable isotope ratio mass spectrometer Thermo Finnigan Delta Plus Advantage following the analytical procedure detailed in Ceburnis *et al*.^11^. The calibration CO_2_ gas was delivered to the mass spectrometer until the isotopic ratio uncertainty was better than 0.15% and three replicates of each sample were analysed. δ^13^C values were corrected for blank and standard HCO_3_^−^ content in sea water using isotope mixing equations.

Chemical speciation analysis was performed by Ion Chromatography (IC), thermal-optical-transmission (TOT) and liquid chromatography/mass spectrometry (LC/MS) with specific details outlined in Claeys *et al*.^17^. Duplicate injections were performed for all filtered extracts and the agreement of 5% was achieved for all ions, except MSA and oxalate (10%).

## Additional Information

**How to cite this article**: Ceburnis, D. *et al*. Stable isotopes measurements reveal dual carbon pools contributing to organic matter enrichment in marine aerosol. *Sci. Rep.* **6**, 36675; doi: 10.1038/srep36675 (2016).

**Publisher’s note**: Springer Nature remains neutral with regard to jurisdictional claims in published maps and institutional affiliations.

## Supplementary Material

Supplementary Information

## Figures and Tables

**Figure 1:**
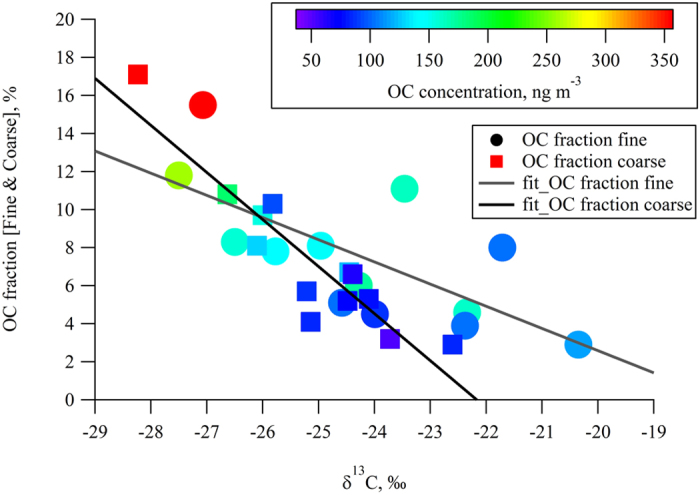
OC fraction vs δ^13^C (HCO_3_^−^ corrected) in fine (<2.5 μm in diameter) and coarse (>2.5 μm in diameter) particles at Amsterdam Island during the austral summer of 2007.

**Figure 2:**
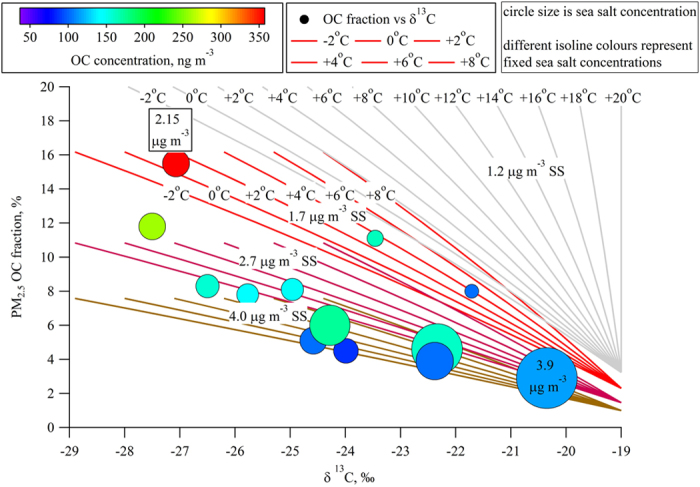
Fractional OC dependence on δ^13^C in the fine (<2.5 μm in diameter) sea spray fraction combined with the simulations of the dual-pool mixing model. The circle size represents sea salt concentration in μg m^−3^ with two actual concentrations noted next-to/inside it; circle colour represents the amount of OC in fine fraction; different colours of isolines represent simulations with fixed sea salt concentrations (light grey – 1.2 μg m^−3^, red – 1.7 μg m^−3^, purple – 2.7 μg m^−3^ and pale brown – 4.0 μg m^−3^); isolines represent carbon isotope ratios of phytoplankton (−30‰ to −25‰) related to sea surface temperatures within Circumpolar Antarctic Circulation (from −2 °C to +8 °C); light grey isolines represent full range of temperatures (from −2 °C to +20 °C) and corresponding phytoplankton isotope ratios (−30‰ to −19‰). Note maximum attainable fractional OC with different sea salt concentrations.

**Figure 3:**
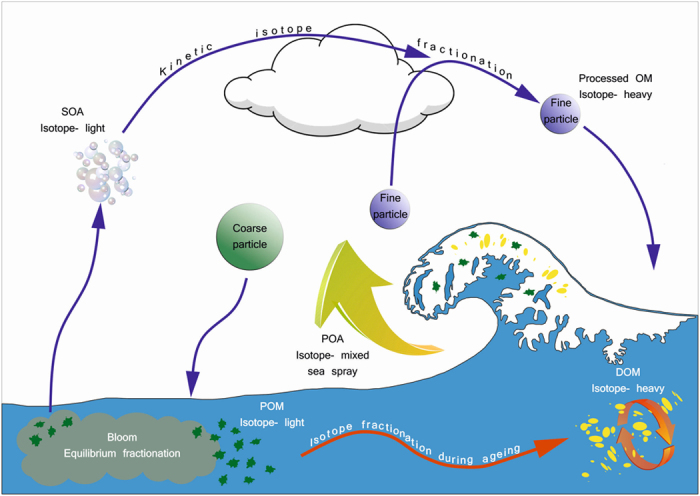
Marine organic matter cycling corroborated by a proposed model. Particulate organic matter (POM) in sea water is produced isotope-light by equilibrium fractionation during photosynthesis and is gradually processed by trophic level interactions in the ocean (weeks-to-years time-scale) to the isotope-heavy dissolved organic matter (DOM). Isotopically mixed sea spray organic matter (POA) undergoes kinetic fractionation by photochemistry and cloud-processing in the atmosphere on a weekly time-scale to isotope-heavy OM. Same scheme applies to secondary organic aerosol (SOA) formed directly or condensed on primary particles. Aged, processed and isotope-heavy OM is returned to the ocean contributing to the continuously recycled and replenished DOM pool. Ultimately the two end-member pools emerge – sequestered, isotope-light and recycled, isotope-heavy OM.
