# Supplementary material for: Stable isotopes measurements reveal dual carbon pools contributing to organic matter enrichment in marine aerosol
Source: Sci Rep. 2016 Nov 7;6:36675. doi: 10.1038/srep36675 (PMC5098177; doi:10.1038/srep36675)
Supplement: Supplementary Information [file srep36675-s1.pdf]

Stable isotopes measurements reveal dual carbon pools contributing to organic matter enrichment in marine aerosol

Darius Ceburnis, Agne Masalaite, Jurgita Ovadnevaite, Andrius Garbaras, Vidmantas Remeikis, Willy Maenhaut, Magda Claeys, Jean Sciare, Dominique Baisnée and Colin D. O'Dowd

Supplementary Material

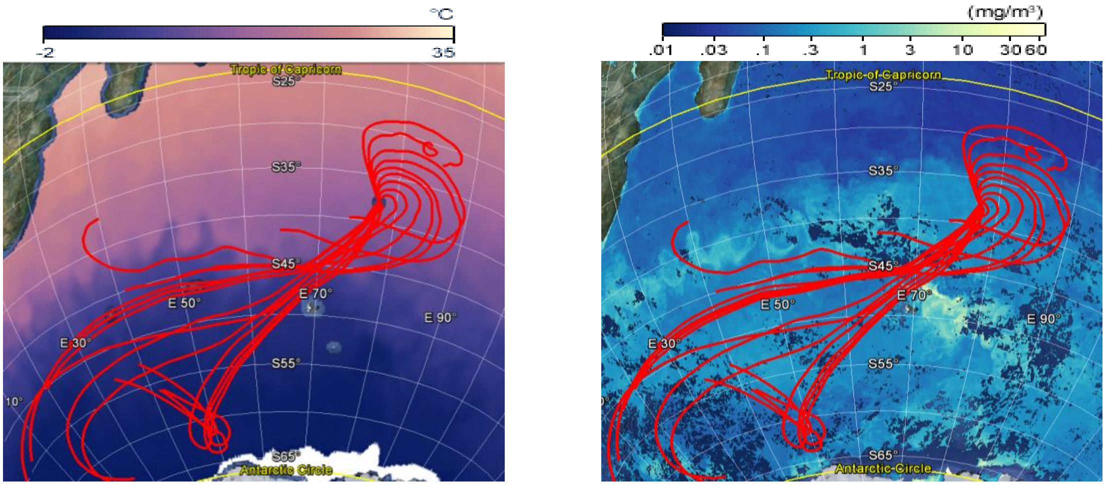

Figure 1. Maps of isobaric air mass back trajectories (every 6 hours for 120 hours at 500 m), generated by NOAA HYSPLIT model and overlaid on: (a) a map of sea surface temperature; and (b) a map of chlorophyll-*a* concentration obtained from NASA Earth Observations (http://neo.sci.gsfc.nasa.gov) for the sampling period 19–24.12.2006 ($\delta^{13}C$ = –26.5 ‰) using "Google Earth" and Map data: SIO, NOAA, U.S. Navy, NGA, GEBCO (2013 Cnes/Spot Image).
